# Supplementary material for: Analysis of technical efficiency of smallholder tomato producers in Asaita district, Afar National Regional State, Ethiopia
Source: PLoS One. 2021 Sep 23;16(9):e0257366. doi: 10.1371/journal.pone.0257366 (PMC8460040; doi:10.1371/journal.pone.0257366)
Supplement: Attachment — Submitted filename: Response to Editor.docx [file pone.0257366.s002.docx]

**Dear Editors,**

I have special gratitude to you, for devoting your valuable time and energy to review my work entitled **“*Analysis of technical efficiency of smallholder tomato producers in Asaita district, Afar National Regional State, Ethiopia (PONE-D-21-15133)*”** and giving constructive comments and invaluable guidance. In a line with this, the author had exhaustively demonstrated and addressed questions and comments raised by editors using point-by-point responses as stated below.

***Point-by-point responses for the questions and suggestions raised by Editor***

1. We note your current Data Availability Statement: “The datasets and articles used to support this study are available from the corresponding author upon reasonable request." However, in the Response to Reviewers you stated: "I have updated the data availability section in the revised manuscript and I have uploaded the minimal data set at supportive file of the revised manuscript." As you have provided a minimal data set file within your manuscript, please clarify this discrepancy between these two statements. Please do note that in the interest of long-term data availability, PLOS data policy does not allow an author to be the sole point of data access. Acceptable restrictions on public data sharing are detailed in the following link:(<https://journals.plos.org/plosone/s/data-availability#loc-acceptable-data-access restrictions>).

**Response:** Thank you dear editor, I have edited such Data Availability Statement on the online submission as “*All relevant data are within the manuscript and its Supporting Information files.”*

2. We also note the file “minimal anonymized data set.dta” in the manuscript in the file named “Revised Manuscript with Track Changes.docx.” Please upload “minimal anonymized data set.dta” as a Supporting Information file.

**Response:** Thank you dear editor, I have uploaded minimal anonymized data set.dta” as a Supporting Information file.

3. Furthermore, please also note that PLOS ONE is unable to publish previously copyrighted maps or satellite images, or images created using proprietary data. For these reasons, we cannot publish images generated by software which copyrights their output (such as Google Maps, Street View, and Earth). In order to use these images in your submission, we require explicit permission from the copyright owner to publish the figures under the CC BY 4.0 license. At this time, please kindly clarify the following regarding Figures 1:
a) Where did the authors obtain the map in Figure 1?
b) Please state whether the map has been previously copyrighted to your knowledge.
c) If the map has been previously copyrighted, we require specific consent from the copyright holder to publish these images in PLOS ONE, under the CC BY 4.0 license. To seek permission from the copyright owner to publish your map figures under the specific Creative Commons Attribution License (CCAL), CC BY 4.0, please contact them with the following text and PLOS ONE Request for Permission form (<http://journals.plos.org/plosone/s/file?id=7c09/content-permission-form.pdf>):

**Response:** Thank you dear editor, I have remove the figure from the revised manuscript.

**Thank you!**
